# Multifunctional Silicone Rubber Nanocomposites by Controlling the Structure and Morphology of Graphene Material

**DOI:** 10.3390/polym11030449

**Published:** 2019-03-08

**Authors:** Ruben Sanchez-Hidalgo, Clara Blanco, Rosa Menendez, Raquel Verdejo, Miguel A. Lopez-Manchado

**Affiliations:** 1 Instituto Nacional del Carbón, INCAR-CSIC, Apartado 73, 33080 Oviedo, Spain; ruben@incar.csic.es (R.S.-H.); clara@incar.csic.es (C.B.); rosmenen@csic.es (R.M.); 2 Instituto de Ciencia y Tecnología de Polímeros, ICTP-CSIC, 28006 Madrid, Spain; rverdejo@ictp.csic.es

**Keywords:** silicone rubber, graphene, mechanical properties, transport properties, nanocomposites

## Abstract

Multifunctional elastomer nanocomposites have been applied in several high-tech fields. The design of materials with tailored properties capable of tuning their performance is a topical challenge. Here, we demonstrate that it is possible to modulate the mechanical and transport properties of silicone rubber nanocomposites by controlling the structure, chemical composition and morphology of the graphene material. Intrinsic graphene properties, such as remaining oxygen groups, specific surface area, and aspect ratio, among others, have a profound effect on the final properties of the nanocomposite. Thus, the thermal conductivity benefits from larger filler size and high aromatic restoration. Whereas mechanical properties and electrical conductivity require a proper balance between filler/polymer matrix interaction and a partial aromatic restoration.

## 1. Introduction

Silicone rubber (SR) is one of the most important synthetic elastomers since it possesses excellent properties, like high elasticity, high thermal stability, resistance to solvents and engine oils, biocompatibility, optical transparency, etc. [1]. Thus, silicone rubber finds use in high-tech applications, such as sealing, gas and aerospace industry, microfluidics, flexible electronics, medical devices, and electrical insulators, among others [2,3,4,5,6]. However, silicone rubbers usually require the use of fillers to improve their mechanical strength and stiffness. Recently, different graphene materials (GMs), thanks to their outstanding properties [7,8,9], have demonstrated to be a potential ideal reinforcement for silicone rubbers owing to its extraordinary mechanical properties and excellent electrical and thermal conductivity [10,11,12,13,14,15,16,17]. Developing high performance rubber nanocomposites using multifunctional fillers is one of the main current industrial challenges, since they combine the unique excellent elasticity of elastomers and different functionalities, as electrical or thermal conductive and permeability properties [18,19]. For instance, Song et al. [10] observed improvements of more than 50% in the thermal conductivity and an increase of about 140% in the tensile strength by addition of 8 wt % of graphene nanoplatelets. Other authors [12,13,14] have demonstrated that the surface treatment of GMs with silane improves the mechanical behavior, thermal stability and thermal conductivity of the composite material, due to a better dispersion and interaction of the graphene with the silicone rubber. Song et al. [15] developed silicone rubber/graphene multilayered films by a layer-by-layer assembly approach, with an exceptionally high thermal conductivity and stretchability. The films exhibited a highly ordered lamellar structure with a preferential orientation of the graphene, which provided continuous thermally conductive pathways. Additionally, the multilayered films with 40 assembly cycles had a thermal conductivity of 2.03 W/mK in the horizontal direction. In a recent study, Wang et al. [17] analyzed the effect of the size of reduced graphene oxides (rGO) sheets on the properties of silicone composites. The authors observed that a middle-sized rGO sheet, of approximately 2.1 µm, had the best mechanical and thermal properties. However, there are no detailed studies concerning the effect of the structure, chemical composition or morphology of the graphene on the physical and mechanical properties of silicone rubber composites.

Our research group has developed a production method that enables the control of different characteristics of graphene materials (GMs), such as specific surface area, oxygen content and lateral size, through the knowledge of the used parent graphite and by tuning the exfoliation/reduction temperatures of the graphene oxide [20,21,22]. In the present study, seven graphene materials were selected to evaluate the role of their structure, morphology and chemical composition on the mechanical behavior and transport properties of silicone rubber composites.

## 2. Materials and Methods

### 2.1. Materials

Commercial synthetic graphite powder (<20 µm Ref. 282863), sulfuric acid (98%), sodium nitrate (>99.8%) and potassium permanganate (<99%) were purchased from Sigma-Aldrich (Darmstadt, Germany). Methyl vinyl silicone rubber under the trade name Bluesil MF135U was kindly supplied by Bluestar Silicones France S.A.S., and bis(1-methyl-1-phenylethyl) peroxide (DCP) was used as vulcanizing agent.

### 2.2. Synthesis of Thermally Reduced Graphene Oxide (TRGO)

The graphite oxide was prepared from commercial graphite following a previously described modified Hummers’ method [20,21]. Briefly, concentrated H_2_SO_4_ (360 mL) was added to a mixture of graphite (7.5 g) and NaNO_3_ (7.5 g) and followed by a careful addition of KMnO_4_ (45 g). The solution was heated to 35 °C and stirred for 3 h. Finally, 3% H_2_O_2_ (1.5 L) was slowly added to the reactor and stirred for 30 min and, subsequently, centrifuged (4000 rpm for 10 min). The remaining solid material was washed with deionized water, centrifuged until neutral pH and vacuum dried. The thermally reduced graphene oxides (TRGOs) were obtained by thermal exfoliation/reduction from graphite oxide at five temperatures, 400, 500, 700, 1000 and 2000 °C as described elsewhere [22,23]. The obtained samples were labeled as TRGO-T, where T is the temperature used in the reduction process.

### 2.3. Preparation of TRGO Filled Silicone Rubber Composites

The composites were prepared in an open two-roll mill (Comerio Ercole S.P.A.) at room temperature. The rotors operated at a speed ratio of 1:1.4. TRGOs at different concentrations (1, 3 and 5 phr (parts per hundred of rubber)) were added to the rubber before incorporating the peroxide. The concentration of DCP in the composite is 0.6 phr. The optimum vulcanization time (Table 3), *t*_90_, was determined by a Monsanto Moving Die Rheometer MDR 2000E. Rubber compounds were then vulcanized at 160 °C and 200 bar of pressure in a thermofluid heated press (Gumix TP300/450/1).

### 2.4. Characterization of Graphene Materials and Their Silicone Composites

The chemical composition of graphene materials (GMs) was evaluated by elemental analysis with a LECO-CHNS-932 microanalyzer and X-ray photoelectron spectroscopy (XPS) on a SPECS system operating under 10^−7^ Pa connected to a MgK_α_ X-ray source (100 W). The C1s peak was fitted using a pseudo-Voigt function.

X-ray diffractograms were recorded in a Bruker D8 Advance diffractometer with a radiation frequency of CuK_α1_ (1.5406 Å) with a power supply of 40 kV and 40 mA. All the XRD patterns were obtained at steps of 0.01 and intervals of 6 s per step. The crystallite sizes along the c-axis (*L_c_*), and a-axis (*L_a_*) were obtained by fitting, respectively, the (002) and (100) reflections using the Scherrer equation [24]. A pseudo-Voigt function was used in order to obtain the best fit of the XRD pattern. The interlaminar distance (*d*_002_) and the estimation of the number of layers (*n*) were obtained from the (002) reflection. s. The number of stacked graphene layers was estimated from *(L_c_*/*d*_002_) + 1.

Raman spectra were recorded on a Renishaw 2000 Confocal Raman Microprobe, (Gloucestershire, UK) from 750 to 3500 cm^−1^ using a 514.5 nm argon ion laser. Five measurements were carried out for each sample. The Raman spectra were normalized and, then, the baseline of the spectrum was extracted using Shirley correction before fitting. The resulting first-order Raman spectra were fitted using two Gaussian functions and three pseudo-Voigt profiles from 800 to 2000 cm^−1^ in five peaks (I, D, D*, G, D’) [20].

The specific surface area was calculated from the N_2_ adsorption isotherms at 77 K using the BET equation. The isotherms were obtained using an ASAP 2020 Micromeritics equipment. The samples were outgassed at 300 °C for 3 h under vacuum prior to the test.

The dispersion degree of TRGO in the rubber compound was analyzed using a TEM JEOL 2000 EX-II instrument operating at 160 keV.

The thermal conductivity of the composites was studied in a stationary flow equipment (LaserComp FOX50) at 25 °C, ASTM C518. The geometry of the samples was square-shaped with dimensions of 25 × 25 × 6 mm^3^.

The impedance measurements of the composites were determined on an ALPHA high-resolution dielectric analyzer (Novocontrol Technologies GmbH) in a frequency range of 10^−1^–10^7^ Hz at room temperature. The geometry of samples was circular with a diameter of 20 mm and a thickness of ≈200 µm. The films were held in the dielectric cell between two parallel gold-plated electrodes. The amplitude of the alternating electric current signal applied to the samples was 1 V. The dielectric response was evaluated by measuring the complex permittivity, ε* = ε’(ω) + jε”(ω) as a function of the frequency (ω). The AC conductivity was measured in the same condition. The DC conductivity (σ_DC_) was calculated using the well-known relation between AC conductivity (σ_AC_) and the frequency (ω) for composite materials [25].

The tensile tests were performed on an Instron uniaxial machine at room temperature, with a cross-head speed of 500 mm/min, following the ASTM D412 specifications. At least six specimens of each sample were tested to obtain a representative mean value of the results.

The rheological study was performed on an RPA 2000 (Rubber Process Analyzer, Ohio, USA) from Alpha Technologies with a deformation of 6.98% and a frequency of 1.667 Hz at 160 °C for 45 min.

The number of active network chain segments per unit of volume (crosslinking density) was determined using solvent-swelling measurements (toluene and n-heptane for 72 h at 30 °C) by application of the Flory–Rhener equation [26].

## 3. Results and Discussion

### 3.1. Characterization of Graphene Materials

Table 1 summarizes the most relevant features of graphene materials used in this study. The morphological and structural changes coming from the thermal reduction have been discussed in detail in previous studies [21,23]. The most significant characteristics arising from the reduction temperature that can influence the properties of the silicone rubber composites are: (i) a gradual increase of the C/O ratio, (ii) a higher restoration of the pristine graphitic 2D-structure, (iii) an increase of the structural order, and (iv) a higher number of layers. On the other hand, the specific surface BET gradually increases with the temperature up to 700 °C, reaching a maximum value of 487 m^2^/g, decreasing drastically at higher temperatures to 161 m^2^/g for TRGO-2000 °C.

### 3.2. Morphology of TRGO Filled Silicone Rubber Composites

**Figure 1:**
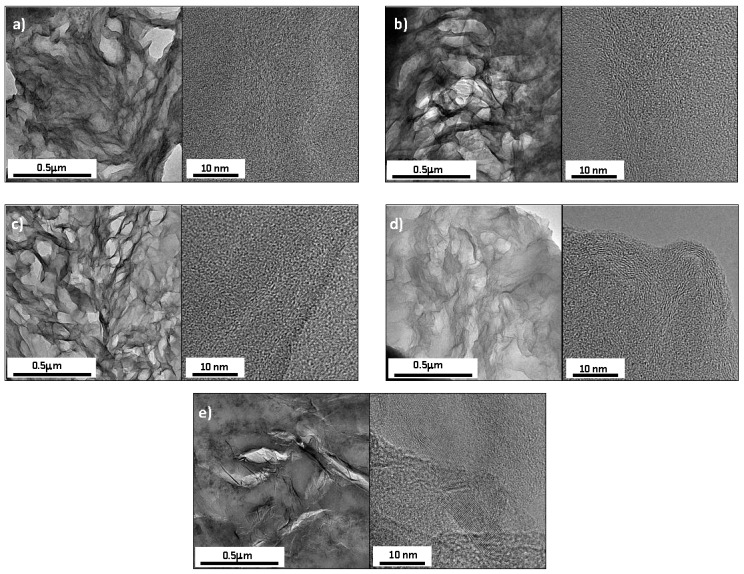
TEM images of thermally reduced graphene oxide (TRGO) filled silicone rubber composites at 5 phr: (**a**) SR/TRGO-400, (**b**) SR/TRGO-500, (**c**) SR/TRGO-700, (**d**) SR/TRGO-1000 and (**e**) SR/TRGO-2000.

Figure 1 shows TEM images of different TRGO filled silicone composites at 5 phr. TRGO reduced to temperatures up to 1000 °C, are homogeneously dispersed in the silicone matrix forming an interconnected network with a small distance between fillers. However, TRGO reduced at 2000 °C shows a worse dispersion with the presence of large aggregates. These aggregates are the result of the reduction temperature, 2000 °C, which almost completely removes the oxygen groups and increases the aromaticity of the graphitic structure and the number of layers (Table 1). This morphology prevents the formation of an interconnected filler structure.

### 3.3. Mechanical Properties of TRGO Filled Silicone Rubber Composites

The mechanical behavior of silicone/TRGO composite materials is shown in Table 2. All TRGOs act as effective reinforcing agents, increasing the value of tensile strength at several deformations, 50%, 100%, 300% and 500%. In general, this reinforcing effect gradually increases with the filler concentration in the composite. However, there are clear differences as a function of the temperature used for reducing the TRGO. It is evident that the structural characteristics and the chemical composition of TRGO have a strong influence on the final mechanical properties of the material. This different behavior of TRGOs is mainly due to the combination of two critical parameters, the specific surface area BET and the oxygen content [27]. Thus, TRGOs-700 and 1000 with larger surface areas, 487 and 467 m^2^/g, respectively, are the most reinforcing fillers since they have a better dispersion in the silicone matrix (Figure 1). Larger surface areas should lead to a greater number of contacts between the filler and the matrix, resulting in stronger interfacial interactions.

Tensile strength at low deformations increases by over 170% and 360% for TRGO-700 and TRGO-1000, respectively, with 5 phr of filler. The greater reinforcing effect of TRGO-1000 can be due to its lower functional group content. During the vulcanization process, the peroxide undergoes a homolytic breakage producing highly reactive alkoxy radicals, which abstract an H-atom from the silicone, and form polymeric-macroradicals. Two macroradicals then combine to form a covalent crosslink. However, there are many secondary reactions that would lead to a decrease in the efficiency of peroxide as a crosslinking agent. Some of these reactions involve the own reorganization of the radical peroxide, abstraction of hydrogen from another donor different from the backbone of the polymer and the presence of oxygen [28]. Therefore, it is necessary to take into account these possible secondary reactions to be able to interpret the mechanical properties of SR/TRGO composites. TRGO-700 with a higher oxygen content, around 8 wt % compared to 1 wt % of TRGO-1000, is susceptible to form secondary reactions parallel to the crosslinking process, such as abstraction of protons from the alcohol of the carboxylic groups [29], or recombination with the epoxy groups [30] present in the basal plane. In fact, the TRGO filled silicone composites with a smaller surface area and higher oxygen content, TRGO-400 and TRGO-500, show the worst mechanical properties, because they partly inhibit the peroxide vulcanization reaction. Hence, the reinforcing effect of TRGO is ascribed to the combination of two factors: the surface area and the number of oxygen groups. TRGO is more effective the larger its surface and the lower the content of oxygen groups on its surface.

**Figure 2:**
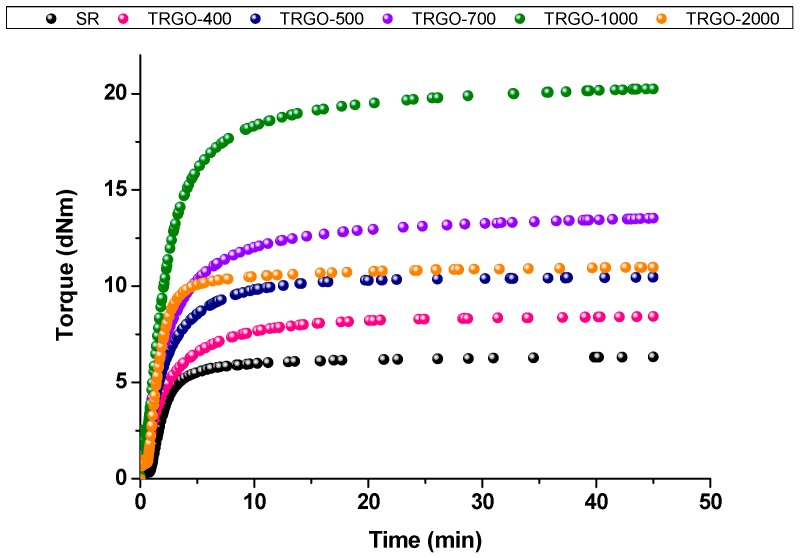
Rheometric curves of TRGO filled silicone composites at 5 phr of filler obtained at 160 °C.

This behavior was corroborated by the rheological analysis carried out to determine the vulcanization curves of the composites (Figure 2). The parameters of the vulcanization reaction are reported in Table 3. The addition of TRGO increases the torque value, this effect being more marked for TRGO-1000. An increase in the torque value is related to a higher cross-linking density, as deduced by swelling measurements (Table 3). Hence, TRGOs with higher surface area and lower oxygen content disperse better in the silicone, forming strong interactions with the rubber matrix.

### 3.4. Electrical Properties of TRGO Filled Silicone Rubber Composites

**Figure 3:**
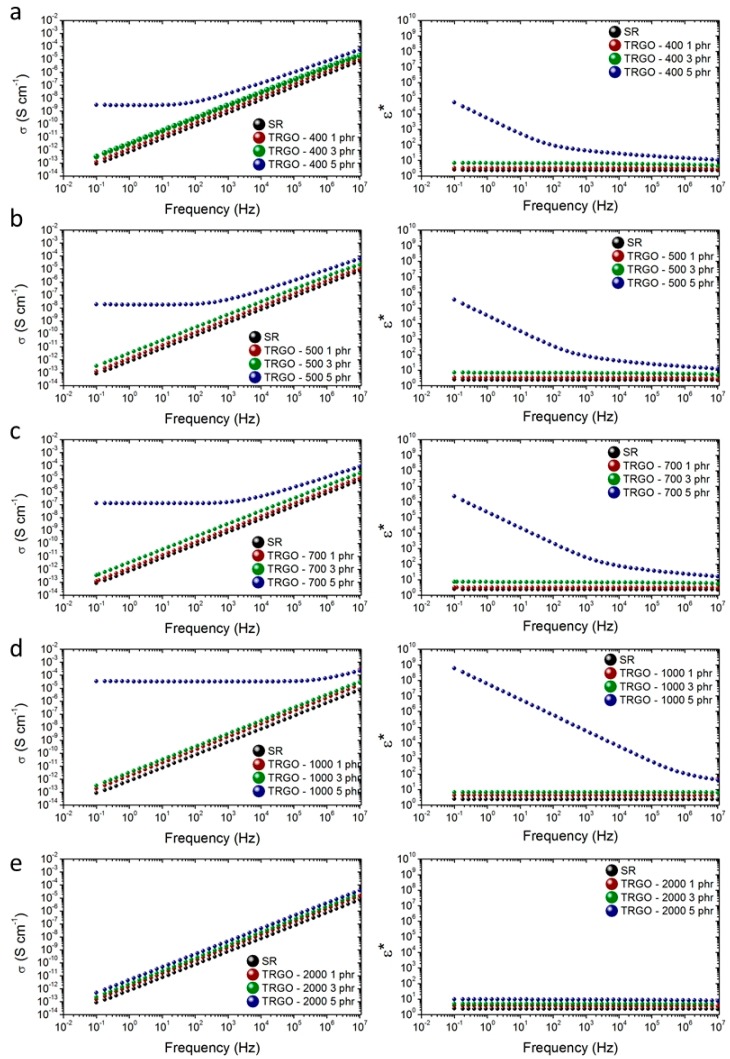
AC electrical conductivity and permittivity for TRGO filled silicone rubber composites at several filler loadings.

The electrical conductivity and permittivity in the frequency domain for TRGO filled silicone rubber composites at different loading fractions measured at room temperature are shown in Figure 3. At low TRGO contents, up to 3 phr, the AC electrical conductivity of the material is frequency independent with slight variations from pristine silicone. However, by increasing the TRGO content in the composite, 5 phr, the electrical conductivity of the material is frequency-dependent, with an increase in the value of the conductivity between four and eight orders of magnitude with respect to pristine silicone. This behavior is typical of a two-phase system commonly described by the percolation theory [31], which indicates that at a certain concentration, fillers form interconnected conductive pathways, reaching the electrical percolation threshold.

In a composite material, the AC conductivity is composed of two terms:

(1) $$\sigma_{DC}^{*}=\sigma_{DC}+A\omega^{s},$$

where $\sigma_{DC}$ is the direct current conductivity, *A* is a pre-exponential factor and *s* is an experimental parameter with values between 0 and 1 [32]. For insulating materials *s* = 1. Hence, from Equation (1), the DC conductivity values can be obtained by extrapolating the broadband AC conductivity to 10^−1^ Hz.

**Figure 4:**
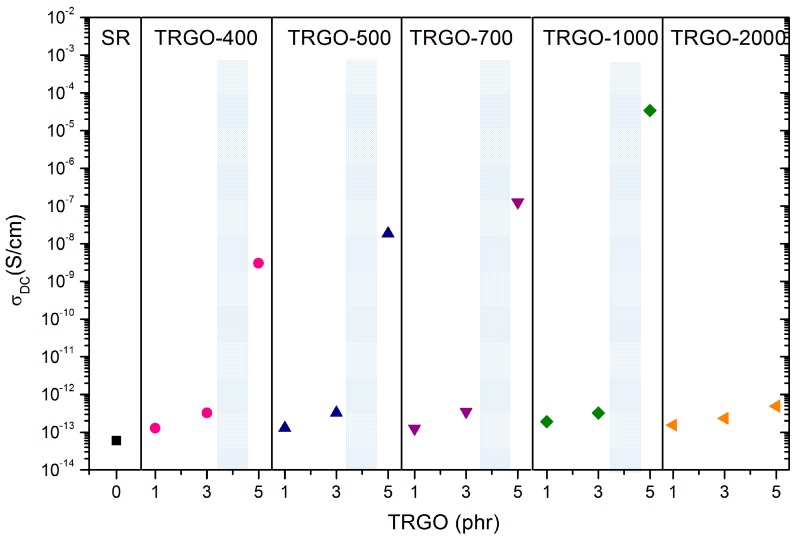
DC electrical conductivity for TRGO filled silicone rubber composites.

Figure 4 shows DC electrical conductivity for the studied silicone/TRGO composites. The dotted lines indicate the electrical percolation threshold for each TRGO. Except TRGO-2000, all TRGOs attain the electrical percolation at loading contents between 3 and 5 phr of filler, and TRGO-1000 at 5 phr exhibits the highest electrical conductivity, with a value of 3.4 × 10^−5^ S/cm, higher than those reported in the literature, even at a lower concentration [10,13]. This behavior can be attributed to the intrinsic structure of TRGO, with a partial aromatic restoration of the basal plane and to its high specific surface area (Table 1). These parameters favor a good dispersion in the silicone matrix, and enable the electrical conduction via the tunneling effect between adjacent graphene layers [33]. Composites filled with TRGOs-400, 500 and 700 show electrical percolation but with lower electrical conductivity values due to their weak aromatic restoration and the presence of numerous defects (oxygenated groups and atomic vacancies) in the basal plane [21].

The composite filled with TRGO-2000, although having the most aromatic restored lattice in the basal plane, does not reach the percolation threshold even at 5 phr nanofiller content. This may be due to its poor dispersion in the silicone matrix (Figure 1), as the result of its low surface area and absence of oxygen functional groups. This absence causes the stacking of the sheets by π-π interactions and a poor interaction with the rubber matrix. A similar behavior was observed in a previous work of the authors, based on TRGO filled epoxy resin composites [23].

On the other hand, the dielectric permittivity hardly varies until the composite material reaches the electrical percolation, where a drastic increase occurs with the frequency. This behavior is associated to the Maxwell–Wagner–Sillars (MWS) effect, typical of composite materials with different dielectric constants [34,35,36]. This effect is associated to the interfacial polarization due to the migration of charge carriers through the phases of the material.

### 3.5. Thermal Conductive of TRGO Filled Silicone Rubber Composites

The fabrication of thermally conductive elastomers is of great interest to be used as thermal interface materials (TIM) to prevent overheating of electronic devices. The incorporation of conductive fillers with a high aspect ratio, such as graphene, is a feasible strategy to prepare thermally conductive rubber nanocomposites. It has been demonstrated that the sheet-like geometry of graphene may have lower interfacial thermal resistance and, thus, produce larger improvements in the thermal conductivity of the polymer composites [37]. Experimental and theoretical studies indicate that any or all factors, including filler type, interfacial bonding, aspect ratio of the fillers, dispersion, and orientation, can affect the thermal conductivity of nanocomposites [38,39]. However, to the best of our knowledge, the effect of structure, chemical composition and morphology of graphene on the thermal conductivity of rubber composite has not been analyzed.

**Figure 5:**
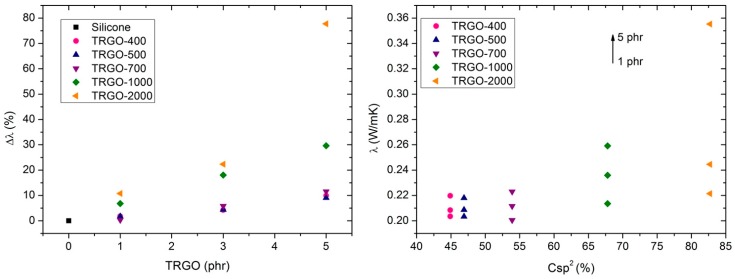
(**left**) Thermal conductivity enhancement of TRGO filled silicone rubber composites at 25 °C and (**right**) variation of the thermal conductivity as a function of Csp^2^ percentage.

The improvement in thermal conductivity of TRGO filled silicone rubber composites at several concentrations is shown in Figure 5 left. The thermal conductivity of the material gradually increases with the TRGO concentration and with the reduction temperature used. TRGO-2000 is the most effective filler, reaching a thermal conductivity value of 0.349 W/mK, which is a 78% increase over pristine silicone. In contrast to the orders of magnitude enhancement in electrical conductivity previously observed, the thermal conductivity of the nanocomposite only exhibits modest improvements, and no percolation transition is observed. This behavior is due to the interfacial thermal resistance, which acts as barrier to heat flow and originates from the mismatch in the phonon spectra of the two phases and possible weak contact at the interface [40].

Unlike other studies, where the functionalization of carbon nanoparticles improved the thermal conductivity of the composite material [14], in our case, the best results were obtained with TRGO-2000, without functional groups in its structure. This increase is associated with the large aromatic restoration and a more compact structure achieved with the heat treatment at 2000 °C. This TRGO exhibits a higher particle size and a greater number of stacked layers, around 19 (Table 1). These characteristics result in the formation of large aggregates that minimizes the coupling losses due to effective diffusion of phonons across the aggregates [41]. Similar results were observed by other authors, who demonstrated that the composites containing large particles have superior thermal conductivity compared to those filled with small particles [42,43,44]. It is evident that there is a close relationship between the percentage of restoration of the pristine graphitic 2D-structure, and the ability of the material to dissipate heat (Figure 5 right).

Although TRGO-1000 showed a better dispersion, as corroborated by TEM (Figure 1), the thermal conductivity of the composite is lower than TRGO-2000 due to the presence of large defects in the basal plane and a higher number of interfaces, which would hinder the correct transition of phonons.

## 4. Conclusions

Multifunctional rubber nanocomposites with performance enhancement were designed using an industrial manufacturing methodology. We have demonstrated that it is possible to modulate the mechanical properties and transport phenomena of TRGO filled silicone rubber composites, controlling the morphological and structural characteristics of TRGO, as well as its concentration in the composite material. Thus, TRGOs with high specific surface area and a partial aromatic restoration are ideal fillers to improve the mechanical properties and provide electrical conductivity to the polymer. Meanwhile, TRGOs with a large particle size and aromatic restoration are the most effective to produce thermally conductive elastomer nanocomposites.

The ability to control thermal conductivity of elastomer nanocomposites is a highly desirable property, which can be exploited in applications that may require a material to be thermally conductive but electrically insulating such as in power electronics, electric motors, etc.

## Figures and Tables

**Table 1:** Composition, and morphological and structural parameters of graphene materials.

	Elemental Analysis (wt.%)						XPS (%)							S_BET_	XRD				Raman Spectroscopy
Sample	C	O	N	S	H	C/O	Csp^2^	Csp^3^	C-OH	C-O-C	>C=O	COOH	π-π*	(m^2^/g)	*d*_002_ (nm)	*L_c_* (nm)	*L_a_* (nm)	*n*	*I*_D_/*I*_G_
Graphite oxide	48.2	49.3	0.1	0.3	2.1	0.98	6.0 ± 1.3	35.8 ± 5.0	6.1 ± 1.2	27.7 ± 2.3	11.5 ± 5.9	13.0 ± 3.6	0.0	45	0.852	9.60	26.20	12	1.12 ± 0.03
TRGO-400	79.1	18.8	0.2	0.4	1.5	4.21	44.9 ± 1.5	25.9 ± 0.6	10.1 ± 0.1	10.5 ± 0.1	3.0 ± 0.1	5.5 ± 0.9	0.0	342	0.357	1.50	7.31	4	1.29 ± 0.08
TRGO-500	81.0	17.4	0.2	0.3	1.1	4.66	46.9 ± 0.4	24.1 ± 0.1	11.1 ± 0.1	9.7 ± 0.4	2.5 ± 0.1	5.7 ± 0.1	0.0	400	0.357	1.75	7.31	4	1.19 ± 0.02
TRGO-700	91.0	8.0	0.3	0.0	0.7	11.38	53.9 ± 0.1	18.5 ± 6.4	10.8 ± 3.0	8.8 ± 1.1	4.8 ± 1.6	3.2 ± 0.9	0.0	487	0.347	1.57	7.83	4	1.20 ± 0.02
TRGO-1000	98.6	1.0	0.0	0.0	0.4	98.6	67.8 ± 0.3	15.1 ± 0.3	11.7 ± 1.2	0.0	4.2 ± 0.2	0.0	1.2 ± 0.1	467	0.360	1.39	8.43	5	1.48 ± 0.05
TRGO-2000	99.7	0.2	0.0	0.0	0.1	498.5	82.7 ± 1.3	10.1 ± 0.3	5.9 ± 1.3	0.0	0.0	0.0	1.3 ± 0.2	161	0.341	6.10	11.56	19	0.18 ± 0.08

**Table 2:** Tensile properties of TRGO filled silicone rubber composites.

Sample		Stress at 50% (MPa)	Stress at 100% (MPa)	Stress at 300% (MPa)	Stress at 500% (MPa)	Maximum Stress (MPa)	Deformation at Break (%)
**SR**		0.46 ± 0.01	0.66 ± 0.02	1.84 ± 0.04	3.74 ± 0.10	7.28 ± 0.77	742 ± 40
**TRGO-400** **(phr)**	**1**	0.50 ± 0.02	0.76 ± 0.02	2.13 ± 0.03	4.09 ± 0.04	6.60 ± 0.75	682 ± 46
	**3**	0.48 ± 0.02	0.73 ± 0.02	1.68 ± 0.05	2.96 ± 0.08	5.43 ± 0.38	772 ± 26
	**5**	0.56 ± 0.01	0.85 ± 0.01	1.72 ± 0.01	2.82 ± 0.03	4.19 ± 0.17	701 ± 27
**TRGO-500** **(phr)**	**1**	0.53 ± 0.01	0.81 ± 0.01	2.27 ± 0.02	4.33 ± 0.06	7.18 ± 0.67	697 ± 39
	**3**	0.83 ± 0.02	1.32 ± 0.02	3.26 ± 0.05	5.47 ± 0.10	6.49 ± 0.40	579 ± 22
	**5**	0.77 ± 0.02	1.19 ± 0.02	2.35 ± 0.02	3.69 ± 0.02	4.12 ± 0.13	559 ± 15
**TRGO-700** **(phr)**	**1**	0.52 ± 0.02	0.79 ± 0.03	2.29 ± 0.06	4.46 ± 0.10	7.25 ± 0.32	689 ± 19
	**3**	1.01 ± 0.03	1.61 ± 0.04	3.85 ± 0.06	6.32 ± 0.03	6.17 ± 0.54	493 ± 36
	**5**	1.16 ± 0.02	1.80 ± 0.01	3.40 ± 0.03	--	3.73 ± 0.14	342 ± 19
**TRGO-1000** **(phr)**	**1**	0.61 ± 0.02	0.94 ± 0.02	2.70 ± 0.03	5.18 ± 0.08	7.28 ± 0.49	633 ± 28
	**3**	0.88 ± 0.04	1.42 ± 0.06	3.77 ± 0.09	6.49 ± 0.09	6.97 ± 0.35	532 ± 23
	**5**	1.80 ± 0.04	3.06 ± 0.06	--	--	6.00 ± 0.21	247 ± 10
**TRGO-2000** **(phr)**	**1**	0.50 ± 0.01	0.72 ± 0.01	1.96 ± 0.04	3.86 ± 0.09	7.01 ± 0.05	710 ± 29
	**3**	0.66 ± 0.02	1.07 ± 0.02	2.95 ± 0.03	5.33 ± 0.03	7.48 ± 0.48	642 ± 29
	**5**	0.74 ± 0.02	1.25 ± 0.02	3.04 ± 0.03	4.98 ± 0.03	5.99 ± 0.21	584 ± 17

**Table 3:** Curing characteristics of TRGO filled silicone rubber composites.

Sample		*S*_min_ (dNm)	*S*_max_ (dNm)	Δ*S* (dNm)	*t*_s2_ (min)	*t*_90_ (min)	*M*c (g/mol)	ν (10^−4^ mol/cm^−3^)
**SR**		0.43	8.03	7.61	1.15	6.65	7305.6 ± 190.5	0.68 ± 0.02
**TRGO-400** **(phr)**	**1**	0.39	7.63	7.24	1.23	7.06	6735.2 ± 284.4	0.74 ± 0.03
	**3**	0.63	7.95	7.32	1.43	13.76	6399.0 ± 744.2	0.79 ± 0.09
	**5**	1.38	8.43	7.05	1.35	10.63	8374.5 ± 55.4	0.60 ± 0.01
**TRGO-500** **(phr)**	**1**	0.37	7.85	7.48	1.29	7.02	6335.0 ± 86.1	0.79 ± 0.01
	**3**	0.63	10.56	9.93	1.03	8.77	4733.7 ± 273.4	1.06 ± 0.06
	**5**	1.58	10.48	8.90	1.14	8.49	5997.2 ± 772.2	0.84 ± 0.11
**TRGO-700** **(phr)**	**1**	0.39	8.31	7.92	1.05	6.87	6508.7 ± 31.6	0.77 ± 0.01
	**3**	0.80	12.44	11.64	0.94	10.00	4048.9 ± 113.5	1.24 ± 0.03
	**5**	1.75	13.53	11.78	0.93	12.31	3981.4 ± 113.5	1.26 ± 0.04
**TRGO-1000** **(phr)**	**1**	0.53	9.67	9.14	1.09	7.83	5949.8 ± 237.5	0.84 ± 0.03
	**3**	0.78	12.54	11.76	0.85	5.95	4701.6 ± 111.5	1.06 ± 0.03
	**5**	1.86	20.25	18.39	0.90	10.40	2342.6 ± 140.1	2.07 ± 0.04
**TRGO-2000** **(phr)**	**1**	0.48	8.55	8.07	1.15	6.34	6097.9 ± 660.5	0.77 ± 0.01
	**3**	0.56	9.83	9.27	0.95	5.75	5290.6 ± 227.1	0.95 ± 0.04
	**5**	0.67	10.99	10.32	1.00	4.51	4701.6 ± 111.5	1.06 ± 0.03
